# Reply to letter: outdated or underrated? the case for low-protein diets in CKD

**DOI:** 10.1590/2175-8239-JBN-2025-0065rpen

**Published:** 2025-10-10

**Authors:** Abdullah Bawazir, Joel M. Topf, Swapnil Hiremath

**Affiliations:** 1 University of Ottawa, Ottawa Hospital, Department of Medicine, Division of Nephrology, Ottawa, ON, Canada.; 2 Ministry of National Guard Health Affairs, King Abdulaziz Medical City, Department of Medicine, Division of Nephrology, Riyadh, Saudi Arabia.; 3 Oakland University, William Beaumont School of Medicine, Rochester, Michigan, USA.

Dear Editor,

We thank Drs. Mafra and Fouque for their interest in our review on low protein diets (LPD) and for the opportunity to clarify our conclusions^1,2^. We address their concerns below and provide clarifications to prevent misinterpretation of our analysis.

We did not “dismiss” older studies outright; rather, we critically appraised their limitations. While early single-center trials report a slower CKD progression with LPD, larger and longer trials have failed to confirm a robust benefit on hard outcomes, a conclusion reinforced by a 2023 Cochrane analysis in diabetic CKD. Mafra and Fouque cite a 2018 meta-analysis as “strong evidence” of benefit, but that meta-analysis pooled many of these same heterogeneous and small-scale trials^3^. We maintain that the modest slowing of progression observed in some studies must be weighed against inconsistent results across the broader evidence base.

Mafra and Foque state that an LPD under expert supervision is safe. Our concern is that even with the guidance from a dietitian, an overly restrictive diet poses risks. In the follow-up of the MDRD study, the LPD group lost significant weight despite isocaloric planning, and in the long-term follow-up, that group had a significantly higher mortality rate compared to controls (39% vs. 23%). The study by Cianciaruso et al.^4^, which reported no increase in malnutrition risk when patients were managed by a nutritionist, also found no difference in renal outcomes between patients on 0.55 g/kg and 0.8 g/kg protein diets, reinforcing our point that more stringent protein restriction conferred no added benefit. Moreover, in practice, many CKD patients do not have consistent access to dietitians, and adherence to LPD is often poor.

Indeed, newer pharmacotherapies should not replace dietary management. Our review highlighted breakthroughs like flozins, which have demonstrated consistent renoprotective effects in RCTs, unlike the LPD trials. Emphasizing such therapies was never intended to “imply dietary efforts are worthless” (as the letter suggests), but to ensure that patients benefit from robust and properly proven measures. We disclosed all potential conflicts of interest, and our enthusiasm for novel therapies is grounded in the strength of evidence, not industry influence. There is no dichotomy here: we favor evidence-based practice, and if LPD demonstrates robust benefits like flozins do, we will be equally enthusiastic about its implementation.

Regarding the specific trial interpretations: we acknowledge that different readers may weigh individual studies differently, but the selective reading of only positive findings is misleading^4,5^. We absolutely agree that avoiding or postponing dialysis is a paramount goal; our emphasis on new therapies is reflects the fact that they robustly accomplish this goal consistently across multiple studies and populations. We share the letter writers’ concern for quality of life including keeping them off dialysis as long as safely possible, but urge equal consideration of the loss of quality of life with an LPD.

Finally, we wholeheartedly endorse the value of multidisciplinary care. If our article’s title, “an outdated strategy”, was perceived as diminishing the role of dietitians, that was not our intention and we are happy to correct. Our critique was aimed at the old paradigm of rigid protein restriction as a one-size-fits-all solution, not at the concept of dietary care itself. In summary, we respectfully stand by our conclusions: there is scant high-quality evidence that strict protein restriction meaningfully slows CKD progression in the modern era, especially compared to the proven impact of newer therapies. LPDs have not lived up to their longstanding promise, and the field of nephrology has advanced with effective therapies. Just as it is important to develop novel therapies, it is equally important to move beyond strategies that have failed to demonstrate durable impact. We appreciate Mafra and Fouque’s commitment to dietary management and agree that when an LPD is chosen, it must be implemented with vigilant nutritional supervision to avoid harm. Ultimately, our shared goal is to maximize both patient longevity and quality of life. In 2025, the best care integrates both nutritional and pharmacological innovations, rather than relying on outdated strategies in isolation.

## Data Availability

There is no original data in this manuscript. All data quoted is from published studies.
