# Supplementary material for: Reply to letter: outdated or underrated? the case for low-protein diets in CKD
Source: J Bras Nefrol. 2025 Oct 10;47(4):e20250065rp. doi: 10.1590/2175-8239-JBN-2025-0065rpen (PMC12533501; doi:10.1590/2175-8239-JBN-2025-0065rpen)
Supplement: Translation

# Translation: Resposta à carta: obsoletas ou subestimadas? O caso das dietas hipoproteicas na DRC

**DOI:** 10.1590/2175-8239-JBN-2025-0065rppt

**Authors:** Abdullah Bawazir, Joel M. Topf, Swapnil Hiremath

**Affiliations:** 1 University of Ottawa, Ottawa Hospital, Department of Medicine, Division of Nephrology, Ottawa, ON, Canadá.; 2 Ministry of National Guard Health Affairs, King Abdulaziz Medical City, Department of Medicine, Division of Nephrology, Riyadh, Arábia Saudita.; 3 Oakland University, William Beaumont School of Medicine, Rochester, Michigan, USA.

Caro Editor,

Agradecemos aos Drs. Mafra e Fouque pelo interesse em nossa revisão sobre dietas de baixa proteína (LPD) e pela oportunidade de esclarecer nossas conclusões^1,2^. A seguir, abordamos as preocupações levantadas e fornecemos esclarecimentos para evitar interpretações equivocadas de nossa análise.

Não “descartamos” estudos mais antigos de forma categórica; ao contrário, avaliamos criticamente suas limitações. Embora ensaios clínicos iniciais, conduzidos em centro único, tenham relatado uma progressão mais lenta da DRC com a LPD, ensaios maiores e mais longos não foram capazes de confirmar um benefício robusto sobre desfechos concretos, conclusão essa reforçada por uma análise *Cochrane* de 2023 sobre DRC em pacientes diabéticos. Mafra e Fouque citam uma metanálise de 2018 como “forte evidência” de benefício, mas essa metanálise reuniu muitos desses mesmos ensaios heterogêneos e de pequena escala^3^. Sustentamos que a modesta desaceleração da progressão observada em alguns estudos deve ser ponderada em relação aos resultados inconsistentes ao longo de todo um conjunto mais amplo de evidências.

Mafra e Fouque afirmam que uma LPD sob supervisão especializada é segura. Nossa preocupação é que, mesmo com a orientação de um nutricionista, uma dieta excessivamente restritiva apresente riscos. No acompanhamento do estudo MDRD, o grupo LPD perdeu peso de forma significativa, apesar do planejamento isocalórico e, no acompanhamento de longo prazo, esse grupo apresentou uma taxa de mortalidade significativamente maior em comparação aos controles (39% vs. 23%). O estudo de Cianciaruso et al.^4^, que não relatou aumento no risco de desnutrição quando o manejo do paciente foi realizado por um nutricionista, também não encontrou diferença nos desfechos renais entre pacientes em dietas com 0,55 g/kg e 0,8 g/kg de proteína, reforçando nosso argumento de que uma restrição proteica mais rigorosa não conferiu benefício adicional. Além disso, na prática, muitos pacientes com DRC não têm acesso consistente a nutricionistas, e a adesão à LPD é frequentemente deficiente.

De fato, as novas farmacoterapias não devem substituir o manejo dietético. Nossa revisão destacou avanços, como as gliflozinas, que demonstraram efeitos renoprotetores consistentes em ECRs, ao contrário de ensaios com LPD. Enfatizar tais terapias nunca teve a intenção de “sugerir que os esforços dietéticos são inúteis” (como indica a carta), mas sim de garantir que os pacientes se beneficiem de medidas robustas e devidamente comprovadas. Divulgamos todos os potenciais conflitos de interesse, e nosso entusiasmo por novas terapias é fundamentado na robustez das evidências, não em influências da indústria. Não há dicotomia aqui: somos a favor da prática baseada em evidências e, se a LPD demonstrar benefícios robustos como os das gliflozinas, teremos o mesmo entusiasmo quanto à sua implementação.

Em relação às interpretações específicas dos ensaios: reconhecemos que diferentes leitores podem atribuir pesos distintos a estudos individuais; contudo, a leitura seletiva apenas dos achados positivos é enganosa^4,5^. Concordamos plenamente que evitar ou postergar a diálise é um objetivo primordial; nossa ênfase em novas terapias reflete o fato de que elas alcançam esse objetivo de forma consistente e robusta em diversos estudos e populações. Compartilhamos a preocupação dos autores da carta quanto à qualidade de vida, incluindo manter os pacientes fora da diálise pelo maior tempo possível e de forma segura, mas pedimos que se considere igualmente a perda de qualidade de vida associada à LPD.

Por fim, endossamos sinceramente o valor do cuidado multidisciplinar. Se o título do nosso artigo, “uma estratégia obsoleta”, foi interpretado como uma forma de diminuir o papel dos nutricionistas, essa não foi nossa intenção e teremos prazer em corrigir tal percepção. Nossa crítica foi direcionada ao antigo paradigma da restrição rígida de proteínas como solução única para todos, e não ao conceito de cuidados alimentares em si. Em resumo, respeitosamente mantemos nossas conclusões: há poucas evidências de alta qualidade de que a restrição proteica rigorosa retarde significativamente a progressão da DRC na era contemporânea, especialmente quando comparada ao impacto comprovado de terapias mais recentes. As LPDs não corresponderam à sua promessa de longa data, e o campo da nefrologia avançou com terapias eficazes. Assim como é importante desenvolver novas terapias, é igualmente fundamental ir além das estratégias que falharam em demonstrar impacto duradouro. Agradecemos o compromisso de Mafra e Fouque com o manejo dietético e concordamos que, quando uma LPD é escolhida, ela deve ser implementada com cuidadosa supervisão nutricional para evitar danos. Em última análise, nosso objetivo comum é maximizar a longevidade e a qualidade de vida dos pacientes. Em 2025, o melhor cuidado deve integrar inovações nutricionais e farmacológicas, em vez de depender isoladamente de estratégias obsoletas.

## Disponibilidade de Dados

Este manuscrito não contém dados originais. Todos os dados citados são provenientes de estudos publicados.
